# Reading Groups as a Health‐Promoting Intervention in Upper Secondary Schools: A Qualitative Study

**DOI:** 10.1111/phn.13428

**Published:** 2024-09-27

**Authors:** Sunniva Solhaug Fjelldal, Anne Clancy, Morten Auklend, Hilde Laholt

**Affiliations:** ^1^ Department of Health and Caring Sciences UiT The Arctic University of Norway Tromso Norway; ^2^ Department of Language and Culture UiT The Arctic University of Norway Tromso Norway

**Keywords:** adolescent health, arts‐based health promotion, health promotion, public health nurse, siblings of children with complex care needs

## Abstract

**Objectives:**

To explore public health nurses’ (PHN) perceptions and experiences of supporting siblings of children with complex care needs (CCNs) by using reading groups as a health promotion intervention in upper secondary schools.

**Design:**

An action research approach using a qualitative design.

**Sample:**

Interviews with 10 PHNs.

**Measurements:**

Thematic analysis.

**Results:**

The analysis resulted in the overarching theme “A much needed health promotion intervention in upper secondary schools,” presented in terms of the following three themes: (1) supporting siblings is important, but resources and established procedures are lacking. (2) Reading groups using fiction have potential as a health‐promoting intervention in upper secondary schools. (3) A realistic collaborative approach is necessary for reading groups to be implemented.

**Conclusion:**

PHNs have no established procedures to support siblings of children with CCNs in upper secondary schools. Reading groups can enable PHNs to reach out and support these siblings. Providing the intervention can be a way of reaching all pupils and thus creating an understanding of the plight of siblings who have a brother or sister with CCNs.

## 1 Introduction

The purpose of this study is to explore the perspectives of public health nurses (PHNs) on using reading groups as a health‐promoting intervention to support siblings of children with complex care needs (CCNs) in upper secondary schools.

There are different dimensions of providing social support to siblings, such as emotional and informational, that can help siblings who find everyday life difficult (Wawrzynski et al. 2021). Schools are a major part of adolescents’ lives, and research shows that teachers and classmates are important sources of support for siblings (Gan et al. 2017; Wawrzynski et al. 2021). Siblings in Norway have the right to receive information and emotional support (Norwegian Health Personnel Act 1999). Section 10a of the Norwegian Health Personnel Act states that Norwegian primary care services are responsible for providing support to siblings who have a brother or sister with CCNs. School health services are an integral part of Norwegian primary care (Norwegian Directorate of Health 2021). PHNs in schools are therefore in a unique position to provide supportive care to siblings. However, recent research reveals a lack of established procedures in school health services in Norway to enable PHNs to support siblings (Bergvoll et al. 2023; Haukeland et al. 2022).

It is estimated that 7%–17% of young people are siblings of a child with CCNs (McKenzie Smith et al. 2018). CCNs are defined as chronic physical, developmental, behavioral, or emotional disorders that require healthcare (Brenner et al. 2018,1647). Siblings living in a family with a child with CCNs can find life difficult and have contradictory feelings of shame, anger, and worries, as well as positive feelings about their ill brother or sister, and can be at risk of developing mental health problems (Dinleyici et al. 2019; Haukeland et al. 2015; Løkkeberg et al. 2020; Lövgren et al. 2016; Woodgate et al. 2016). It is therefore important that siblings receive support (Bergvoll et al. 2023; Hartling, Milne, and Tjosvold 2014; Haukeland et al. 2020; Nygård, Clancy, and Kitzmüller 2023; Wolff, Magiati, and Roberts 2023).

## 2 Background

### 2.1 The School Health Service

Norwegian school health services are mandated by law (Norwegian Health Care Act 2011) and are available to all pupils in primary (6–12 years), lower secondary (13–16 years), and upper secondary schools (16+ years). PHNs have office hours at schools and provide drop‐in services as well as standardized universal services for all pupils (Norwegian Directorate of Health 2021). The standardized universal services involve health promotion and illness prevention for all pupils. This includes responsibility for the Norwegian childhood immunization program, screening programs, monitoring physical development, and providing individual and group health dialogues (Norwegian Directorate of Health 2021).

Health dialogues can be a structured discussion between the PHN and pupils following guidelines or open‐ended conversations to discuss matters concerning pupils’ health and well‐being (Laholt et al. 2017; Norwegian Directorate of Health 2021). In health dialogues, PHNs provide guidance and counseling on family health and topics such as nutrition, mental and physical health, puberty development, and sexual health (Norwegian Directorate of Health 2021). However, these dialogues are standard services only in primary and lower secondary schools. PHNs in upper secondary schools do not provide systematic health dialogues but are required to promote health at the individual and group levels to develop an environment that benefits the health of all pupils.

Health‐promoting interventions in schools have the potential to promote the health and well‐being of all adolescents (Das, Salam, and Lassi 2016; O'Reilly, Svirydzenka, and Adams 2018). Collaboration between teachers and PHNs in upper secondary schools is essential for health promotion at individual and group levels, and PHNs can contribute to the teaching of health‐related topics in classes (Norwegian Directorate of Health 2021). Recent research shows that school nurses value collaboration with teachers, where school nurses and teachers complement each other in health‐promoting work for adolescents (Hilli and Pedersen 2021). However, research also reveals that school nurses face challenges in their work due to time and resource constraints (Dahl et al. 2022; Lineberry, Whitney, and Noland 2018), which can affect their capacity to promote health in schools (Hoekstra et al. 2016).

### 2.2 Reading Groups as a Health‐Promoting Intervention

The World Health Organization recommends arts‐based interventions to promote health (Fancourt and Saoirse 2019). Public health approaches have increased the emphasis on the arts in promoting health (Longden, Davis, and Billington 2015), and creative arts‐based interventions have been shown to promote health and well‐being (Clift 2012; Fancourt and Saoirse 2019), such as reading fiction (Kurtts and Gavigan 2017; Lucas and Soares 2013).

Shared reading (Davis 2009; Longden, Davis, and Billington 2015) and bibliotherapy (DeVries and Sunden 2019; Lucas and Soares 2013) are both methods of reading fiction in groups, where one of the aims is to show how fiction can help readers make sense of complex experiences by identifying and exploring issues raised in a work of fiction. Reading fiction aloud in groups creates a space for individuals to share their personal reactions to the text (Davis 2009; Longden, Davis, and Billington 2015). Reading is an activity with the potential to evoke emotional responses regardless of the topic (Lauritzen 2019). A reading group can entail being confronted with different reactions and emotions from peer readers, which under other circumstances could be embarrassing, but this is possible in reading groups, which are often perceived as a free space (Christiansen 2021). Participants in a reading group can discuss sensitive topics without disclosing personal information (Lauritzen, Antonsen, and Nesby 2021; Longden, Davis, and Billington 2015). Shared reading is a method that fosters environments where participants can connect with both the text and each other. In contrast, bibliotherapy typically focuses on the therapeutic benefits of fiction for groups, often emphasizing self‐help (Longden, Davis, and Billington 2015; Lucas and Soares 2013).

Research has shown that reflections on fictional characters can increase readers’ ability to view and understand their personal experiences and emotions when they identify with the characters in a text (Davis 2009; DeVries and Sunden 2019; Lucas and Soares 2013). This process can promote the development of life skills (McCulliss and Chamberlain 2013) and help adolescents understand and reflect on social and emotional issues (McCulliss and Chamberlain 2013). However, a recent Norwegian study showed that fiction is only used in 1% of upper secondary schools as a health promotion intervention for siblings (Bergvoll et al. 2023).

Reading groups can provide the benefit of a supportive environment where pupils can engage with fiction to explore complex emotional and social issues. The health promotion perspective of PHNs is important in the development of school‐based reading groups.

#### 2.2.1 Aim

The main purpose of this research was to explore PHNs’ perceptions of using fiction in reading groups as a health promotion intervention for siblings in upper secondary schools.

## 3 Methods

### 3.1 Design and Sample

This study is part of an action research project exploring the implementation of reading groups in upper secondary schools to support siblings and enhance pupil well‐being in general. Utilizing a qualitative approach, the action research project progressed through four phases: (1) assessing PHNs' current practices to support siblings, (2) gathering PHNs' insights on reading groups as a health‐promoting intervention (the focus of the current study), (3) implementing reading groups, and (4) conducting focus groups with pupils to evaluate their experiences.

This study enabled a thorough exploration of how reading groups can serve as arts‐based health interventions, providing valuable data to inform practice development. By using methods such as semi‐structured interviews (Krueger and Casey 2015), we gathered rich and detailed data that provided insights into PHNs’ perceptions of reading groups as a health promoting intervention. This data are crucial for understanding the possibilities and challenges of how reading groups could function as supportive environments for siblings and other pupils. Furthermore, the qualitative approach facilitated the active involvement of PHNs as stakeholders in the research process. Participant involvement is essential for action research (McNiff 2017).

The selection criteria for this study were that participants were qualified PHNs and that they had experience in the school health service. Email invitations were sent to PHNs in ten municipalities in northern Norway. Three of these emails received no response, and PHNs from two municipalities declined to participate. We received a positive response from PHNs in five municipalities, resulting in 10 PHNs who agreed to participate in the study. All participants provided informed voluntary consent in accordance with the Declaration of Helsinki (World Medical Association 2013).

### 3.2 Data Collection

Data were collected from one focus group discussion (FGD) (*n* = 5), two dyadic interviews (DI‐1 and DI‐2), and one individual interview (II), conducted in May and June 2022. The PHNs’ experience ranged from 1 to 36 years. All the PHNs worked in school health services and eight of the PHNs had experience as school nurses in upper secondary schools. The PHNs represented urban (population >20,000) and rural (population <2000) municipalities (Statistics Norway 2020). Specific details of the participants will not be provided due to ethical considerations related to confidentiality.

To enhance understanding of PHNs’ perceptions, we asked how PHNs saw their role in using fiction and organized reading groups in collaboration with teachers in upper secondary schools. We used the same semi‐structured interview guide (Table 1) in all interviews and follow‐up questions were asked based on the participants’ responses. The interview guide was designed based on discussions between all four authors and in accordance with the study's aim. The first, second, and last authors are PHNs, while the third author is a literary scholar. A pilot study was conducted by the first author, where the participants were two experienced PHNs who had worked in the school health service. This resulted in minor changes to the interview guide.

**TABLE 1:** Interview guide.

	Questions	Probe
**Opening question**	The researcher started by asking the participants their name, their general experience as a PHN, and their experience in the school health service.	Name and experience from school health services.
**Introduction**	What are your experiences of working to promote health aimed at siblings of children with CCNs?	PHNs’ experience in health promotion work with siblings. Do they have interventions and systematic practices in identifying and supporting siblings of children with CCNs? Any challenges?
**Key questions**	What are your experiences of using fiction in the school health service?	PHNs’ experience of using fiction in school health services. Any opportunities/challenges?
	How do you think PHNs can use fiction to support siblings of children with CCNs?	PHNs’ perspectives on using fiction to support siblings of children with CCNs. Any opportunities/challenges?
	Imagine that you as (a) PHN(s) in collaboration with a teacher for an entire class in upper secondary school want to use fiction and reading groups to support siblings of children with CCNs. What do you think the role of the PHN would be in this collaboration?	PHNs’ role in reading groups for an entire class as a health‐promoting support for siblings of children with CCNs. What are the PHNs’ perspectives?
	How do you think the collaboration between PHN and the teacher should be to succeed with such a project?	PHNs’ role in collaboration with teachers in reading groups. Any opportunities/challenges/ethical considerations?
	What do you think is the reason why PHNs use more fiction in primary schools than in upper secondary schools?	Fiction and health promotion in upper secondary schools versus primary schools. PHNs’ opportunities and challenges in using fiction in upper secondary schools.
**Rounding off**	Which competencies do you think PHNs can contribute to reading groups as a health promotion project?	How to establish reading groups successfully—what do we need to think about?
	The participants were asked if there was anything else they would like to add.	Did I miss something important?

Abbreviations: CCNs, complex care needs; PHN, public health nurse.

The FGD was conducted at a health center in the municipality where the PHNs were employed. The first author was the moderator, and the third author was the assistant moderator. The first author conducted the dyadic and individual interviews online using the video conferencing tool Microsoft Teams (UiT The Arctic University of Norway 2023). The interviews lasted between 57 and 74 min and were recorded on a manual voice recorder and a digital voice recorder using a platform (University of Oslo 2022) for online data collection. The study was approved by the Norwegian Agency for Shared Services in Education and Research (Project No. 411733).

### 3.3 The Data Analysis Process

We analyzed the data to identify themes using Braun and Clarke's (2022) six phases of reflexive thematic analysis (Table 2). Phase 1 consisted of familiarization with the dataset, where the first author listened once to the interviews from the recordings before transcribing all the interviews verbatim. The transcripts were carefully read by all the authors. The first author read and re‐read the dataset and took notes on any insights related to the data. These notes were then discussed by all authors. In Phase 2, the first author generated codes and worked systematically through the transcribed interviews, capturing meaning relevant to the aim of the study. Examples of the initial codes were: “Siblings are not part of the healthcare system, unlike sick children,” “No system for identifying siblings,” “Collaboration with a teacher can help to identify siblings,” and “Reading fiction together can provide a sense of fellowship.” However, coding was not a linear process, and discussions with the other authors resulted in some recoding. In Phase 3, the initial themes were identified by the first author, capturing shared patterns of meanings across the dataset. The first author used different colors on the codes that helped to identify patterns that resulted in initial themes. In Phase 4, the initial themes were reconstructed through discussion by all authors and were then developed and reviewed. The first author checked whether the themes made sense in terms of the initial themes, codes, and the full dataset. Subsequently, all authors discussed whether the themes highlighted the patterns from the dataset in relation to the aim of the study. We then started defining and naming the themes in Phase 5 of the analysis process. The first author suggested names for the themes, and all authors discussed these by critically reflecting on questions such as “What story does this theme tell?” (Braun and Clarke 2022). This process resulted in the overarching theme “A much needed health promotion intervention in upper secondary schools.” We then wrote up the results (Phase 6), which are presented in this article, with a story about the dataset that addresses this study's aim. The first author wrote daily notes during the analysis and reports from all discussions in the research team. Each written step of the analyzed data, notes, and reports was stored in a secure database to which all authors had access. Ethical guidelines, in accordance with the Declaration of Helsinki, were followed throughout the research process (World Medical Association 2013).

**TABLE 2:** Overview of the data analysis process.

Overarching theme	A much needed health promotion intervention in upper secondary schools		
Themes	Initial themes	Codes	Quotes
Supporting siblings is important, but resources and established procedures are lacking	PHNs are unaware of the existence of these siblings	When contact, PHNs offer support Must know of the sibling to offer support It is important that siblings are “seen”	*“(…) the way it is in upper secondary school, it's mostly the pupils who contact you (…), that's my experience (…).”* (PHN 8 in DI‐1)
	PHNs have limited resources in upper secondary schools	Lack of time and resources The school health service should be organized to do more health promotion work PHNs practice individual‐oriented work	*“(…) the idea that we should kind of contribute to the school (…) by teaching and doing other things the school needs (…) I do think it's something we should be doing, and perhaps there should be more of a focus on it, but it's kind of a question of resources too (…).”* (PHN 9 in DI‐1)
Reading groups using fictional texts have potential as a health‐promoting intervention in upper secondary schools	Knowledge of using fiction as a health promoting tool is needed	PHN's limited experience of using fiction in their practice Fiction can help to show that the reader is not alone Fiction can give support without making a big issue of the problems	*“I think there must be a list that, you know, healthcare staff (…) have looked at the texts, and decided that they're relevant and useful (…).”* (PHN 5 in FGD)
A realistic collaborative approach is necessary for reading groups to be implemented	A nuanced and flexible approach to using fictional texts	Fictional text representing different narratives The nuances of the sibling perspective	*“(…) relationships between brothers and sisters vary a lot, families are different, relationships between siblings are different (…).”* (PHN 8 in DI‐1)
	The importance of collaboration	PHNs’ framework and resources must be planned Cooperation with the teacher is essential for conducting reading groups Ethical considerations	*“I think if you're going to do it with a teacher, it's really important to sit down together and plan properly (…) what you're going to say something about (…) and then you can use each other, you agree about who will say what (…) of course we have the healthcare expertise that a teacher doesn't have (…).”*(PHN 9 in DI‐1)

Abbreviations: DI‐1, dyadic interview 1; FGD, focus group discussion; PHN, public health nurse.

## 4 Results

The analysis of the interviews with the PHNs resulted in the overarching theme “A much needed health promotion intervention in upper secondary schools”. The findings are presented under the following three themes: (1) supporting siblings is important, but resources and established procedures are lacking. (2) Reading groups using fiction have potential as a health‐promoting intervention in upper secondary schools. (3) A realistic collaborative approach is necessary for reading groups to be implemented.

### 4.1 Supporting Siblings Is Important, but Resources and Established Procedures Are Lacking

#### 4.1.1 PHNs Are Unaware of the Existence of These Siblings

The PHNs spoke about the importance of supporting siblings of children with CCNs, and how they play an important role in identifying siblings in need of support. However, PHNs do not often initiate contact with these siblings. It is often the siblings themselves, teachers, or parents who contact the PHN. The PHNs found it difficult to provide support for siblings when unaware of their existence. Siblings often visit PHNs with other challenges such as finding life difficult, feeling sad, having problems at school, or having minor health complaints. After several visits in the form of drop‐in health dialogues, it may emerge by chance that the adolescent has a brother or sister with CCNs. As one PHN said:

*“(…) after many conversations, now they've told me they're having some problems at home because they have a brother or sister who's (…) ill.”* (PHN 3 in FGD)

The PHNs stated that there were no procedures for identifying the number of siblings of children with CCNs. A PHN explained:

*“(…) Occasionally I get a call from the hospital (…), where I know the parents may have been admitted, or they have some illness (…) but I never get a call from the GP, who often has all the brothers and sisters.”* (II)

The possibility to offer siblings support could also vary according to the PHNs’ involvement in the family, or the size of the municipality. As one PHN explained:

*“(…) there's no system so you can find out about these children, you won't necessarily be involved, it's kind of more by chance when it does happen (…).”* (PHN 8 in DI‐1)

PHNs reported practicing interdisciplinary collaboration with other health professionals concerning children with an illness, yet this did not apply to siblings. The PHNs acknowledge an increasing awareness of children and young people with sick siblings. However, specific measures to reach out to them had not been implemented. They expressed uncertainty about what their role for siblings in school health services should entail. The nurses found that these siblings were not on the agenda in their practice, as one said:

*“(…) it hasn't been discussed in most of the practice I've had. So it hasn't been talked about at all. There hasn't been any focus on (…) brothers and sisters (…).”* (PHN 5 in FGD)

#### 4.1.2 PHNs Have Limited Resources in Upper Secondary Schools

The PHNs in this study were eager to provide support to siblings but were also critical of the fact that supporting siblings involved resources that they did not have. As one said:

*“(…) we don't have these talks for first year pupils that we've been talking about for many years, because we can't see how we can fit them in (…).”* (II)

Some PHNs spoke about not having enough time to carry out existing health promotion interventions at the group level. Due to the scope of their work, PHNs lacked the resources to address the healthcare needs of all pupils and to work across professional boundaries. Some PHNs stated that many individual pupils required advice and guidance, and much of their working hours were spent on individual health dialogues on issues concerning their mental health and wellbeing.

The PHNs added that teachers contacted them if they had pupils they were worried about and the PHNs felt obliged to help those pupils. In such cases, the health dialogues often dealt with mental health problems. Some PHNs felt that if they focused mainly on problems, there would be very little time left for health‐promoting initiatives for other pupils and siblings, as one explained:

*“(…) And I know that I very often fall into the trap, you know, that we focus so much on everything that's painful and difficult, it's like THAT is what we have to work on. But the health promotion part, that kind of gets swept under the carpet when we think we have to limit the extent of the harm and fix what's difficult instead of promoting what's good, looking at what's healthy. And it's actually quite a big part of our job to promote health, to look at what's healthy in people (…). Maybe we need to focus on that even more in our work with siblings (…).”* (PHN 6 in DI‐2)

### 4.2 Reading Groups Using Fiction Have Potential as a Health‐Promoting Intervention in Upper Secondary Schools

#### 4.2.1 Knowledge of Using Fiction as a Health Promoting Tool Is Needed

The PHNs expressed varied personal experiences while reading fiction; it could, for example, enable reflections that promoted an understanding of the different perspectives of being human. Further, a sense of connection through reading alleviated certain feelings such as the feeling of loneliness, as the PHN in the individual interview said:

*“(…) You're never lonely, you're never alone if you like reading. That's the way I feel, just diving into a book that grabs your interest, where you get to know different personalities, where you can recognize yourself, and you can maybe recognize your friends (…).”* (II)

The PHNs in the focus group thought that it was possible to incorporate the use of fiction and reading groups into their practice. Fiction could be used as a health‐promoting intervention for siblings in individual health dialogues and in reading groups in collaboration with teachers. The PHNs stated that they must be adequately prepared and have the necessary knowledge about reading groups and relevant texts, as one of them said:

*“I think there must be a list that, you know, healthcare staff (…) have looked at the texts, and decided that they're relevant and useful (…).”* (PHN5 in FGD)

### 4.3 A realistic Collaborative Approach Is Necessary for Reading Groups to Be Implemented

#### 4.3.1 A nuanced and Flexible Approach to Using Fiction

The PHNs expressed the need for a flexible approach to the introduction of reading groups in collaboration with teachers in upper secondary schools and that the selection of fiction required careful considerations and ethical awareness. Reading groups in collaboration with teachers could lead to discussions on what it is like to live in a family with a chronically ill child. They saw the importance of allowing pupils to participate in reading groups without having to divulge if they have a brother or sister with CCNs. They also stated that fiction should represent different narratives. As one PHN explained:

*“I think relationships between brothers and sisters can vary a lot (…) so in spite of the literature that kind of makes an issue of this (…) it may not legitimize exactly the feelings or the experiences of a particular child (…) you have to realize that this isn't (…) something all siblings experience (…).”* (PHN 8 in DI‐1)

The PHNs also expressed that texts should be short to ensure that pupils would read them and that PHNs could be prepared in advance and have time to read them. The PHNs stated that using fiction in groups could help them gain greater insight into the thoughts and reflections of adolescents. Two of the nurses spoke about the challenges in rural areas with fewer pupils and the need for a different approach that involved whole classes. As one nurse said:

*“I think maybe it's especially important that those of us who come from rural areas, it's really hard to bring together a whole group of brothers and sisters (…) then you might get a group of five with an age range from 3 to 18 (…). It's pretty obvious that it would be a very strange group, but you have to think differently then (…).”* (PHN 6 in DI‐2)

#### 4.3.2 The Importance of Collaboration

The PHNs emphasized the importance of collaborating with teachers. The school library and librarians were also mentioned as important partners, but they lacked established routines for collaboration. However, they saw teachers as playing a natural role in using reading groups as part of Norwegian language classes, where school nurses could contribute and use their expertise as health professionals. One of the PHNs explained the importance of collaboration in this way:

*“(…) so my role as a public health nurse in a project could be this or that (…) so then I can take this or that health perspective. (…) It's about choosing books (…) which books we should have (…), so that we get to be participants. That means that all parties become involved in this project (…) and we plan it together. I feel I must be involved right from the beginning, I'm not going to be invited to join (…) Yes, I should have some ownership of the project too.”* (II)

The PHNs emphasized the importance of pupils being familiar with their school nurse, as one nurse stated:

*“(…) And I think it might be a good idea for us school nurses to be available for a while afterwards in case somebody thinks of something they want to ask about (…), like you do that on a day when you're at school, then you say today you'll be here from this time to that time, and if there is anything, if there's something on your mind that you just want to ask about (…) you can come to my office that's in room number whatever.”* (PHN 7 in DI‐2)

They also highlighted the crucial role of teachers, suggesting that they should inform pupils how to contact the PHN if they experience any emotional reactions during or after the reading groups.

## 5 Discussion

The findings reveal that PHNs considered reading groups using fiction to have potential as a health promotion intervention to support siblings. The use of reading groups in collaboration with teachers could help peers to reflect on what it is like to be a brother or sister of a child with CCNs without revealing any siblings’ private details. However, it is necessary to use a flexible approach to enable reading groups to be implemented and fit each PHN's schedule in collaboration with the teacher. Ethical considerations are important, and PHNs should be available to pupils if needed, throughout and after the reading groups.

### 5.1 Reading Groups as a Possible Health‐Promoting Intervention to Support Siblings in Upper Secondary Schools

PHNs reported that siblings often remain unnoticed unless they initiate contact, making it difficult to provide support without awareness of their needs. Our findings are consistent with previous research indicating that PHNs are resource‐constrained (Lineberry, Whitney, and Noland 2018) and that PHNs work mostly at an individual level (Dahl et al. 2022). Despite legal mandates (Norwegian Health Personnel Act 1999), there is a lack of systematic support for siblings, underscoring the need for targeted health interventions (Bergvoll et al. 2023; Hartling, Milne, and Tjosvold 2014; Haukeland et al. 2020; Nygård, Clancy, and Kitzmüller 2023; Wolff, Magiati, and Roberts 2023). Reading groups emerge as a promising health‐promoting intervention to support siblings, blending healthcare and educational roles between PHNs and teachers. However, PHNs expressed the need for knowledge on using fiction in health promotion. We found that PHNs viewed their role as related to healthcare aspects, while teachers naturally took on an educational role in conducting reading groups.

By involving PHNs in reading groups, the intervention ensures that health support is readily available. PHNs can facilitate health dialogues and offer support tailored to the needs of siblings and other pupils. However, the PHNs mentioned ethical considerations regarding the selection of texts and the ability to safeguard siblings’ privacy in the groups. Research has shown that the emotional experiences of siblings of children with rare disorders are not easily shared in sibling support groups (Haukeland et al. 2015). Reading groups can, however, promote an empathic understanding and different views of life story narratives without revealing personal information (Killick and Bowkett 2015; Lauritzen, Antonsen, and Nesby 2021; Longden, Davis, and Billington 2015; McCulliss and Chamberlain 2013). Understanding siblings’ risk of developing mental health problems (Dinleyici et al. 2019; Haukeland et al. 2015; Løkkeberg et al. 2020; Lövgren et al. 2016; Woodgate et al. 2016) underscores the importance of health promotion interventions in schools to support these siblings. Reading groups can be doubly beneficial for siblings by providing a space to explore and express their feelings and thoughts through fiction. Additionally, the presence of attentive peers who learn about the experiences of being a sibling can offer supportive effects for siblings (Nygård, Clancy, and Kitzmüller 2023). Reading groups could be particularly beneficial in rural areas, where forming support networks is challenging. Recent research from Norway also indicated regional differences in the support provided by PHNs to siblings of children with CCNs (Bergvoll et al. 2023).

However, the effectiveness of reading groups depends on various factors, including individuals’ willingness to engage and their personal relationship to the text. Reading groups can also lead to judgmental and strong reactions towards other group members, which can promote tensions, such as unanswered questions about oneself or others and a sense of being different (Christiansen 2021; Longden, Davis, and Billington 2015). PHNs can play an important role as health professionals who can collaborate with teachers (Hilli and Pedersen 2021) in the selection of texts, contributing their knowledge of health promotion and illness prevention (Dahl et al. 2022), and providing support if necessary. Siblings have a higher risk of poor well‐being (Dinleyici et al. 2019; Løkkeberg et al. 2020) and family life and adolescent health are considered sensitive topics (Powell et al. 2018). Ethical considerations such as vulnerability are important when collaborating on health promotion interventions for adolescents (Hilli and Pedersen 2021; Laholt, McLeod, and Guillemin 2018).

Arts for health initiatives appear to have public health benefits and are cost‐effective (Clift 2012; Fancourt and Saoirse 2019). However, there is a need to emphasize PHNs’ boundaries and involvement in terms of their role in reading groups. This study highlights a possible arts‐based support intervention for siblings in collaboration between school health services and schools. This is in accordance with the World Health Organization's recommendation to strengthen structures for collaboration between the cultural and health sectors and to develop interventions that encourage engagement with arts to support health and well‐being (Fancourt and Saoirse 2019). The proposed reading group intervention has the potential to provide support to siblings, enhance understanding among peers, and integrate health services into the educational framework.

## 6 Strengths and Limitations

This study provides in‐depth knowledge of PHNs’ perspectives on supporting siblings in upper secondary schools and the use of reading groups as an arts‐based intervention. Owing to the COVID‐19 pandemic, it was challenging to recruit PHNs for FGDs. This resulted in a combination of one individual interview (Kvale and Brinkmann 2015), two dyadic interviews (Morgan et al. 2013), and one FGD (Krueger &Casey 2015). This can be considered a limitation of this study. However, the combination of different interviews gave valuable contrasting perspectives and rich data (Kvalsvik and Øgaard 2021; Morgan and Hoffman 2018). We used the COREQ 32‐item checklist (Tong, Sainsbury, and Craig 2007) to validate the study process. The research team consisted of three female PHNs and a male literary scholar, which was seen as a strength in the preparation of the interview guide, during the interview process, and in the data analysis, helping to broaden interpretations and clarify major themes.

## 7 Conclusions

PHNs’ health promotion work in upper secondary schools is characterized by a lack of resources and busy schedules; therefore, our findings show the importance of implementing guidelines at the system level to promote support for siblings in upper secondary schools. Our study shows that reading groups in collaboration with teachers can give PHNs a unique position to promote health for siblings, which can supplement individual health dialogues. Reading groups can raise classmates’ awareness of what it is like to live in a family with a brother or sister who has CCNs, without potential siblings being exposed. However, ethical considerations are important when planning and implementing reading groups in collaboration with teachers. These results are particularly valuable in countries with similarly organized school health services. Reading groups can explore various health topics through fiction, serving as a potential health intervention for other vulnerable groups and as a universal health‐promoting intervention for all schoolchildren. Additionally, this intervention offers potential in educational settings for nurses, enabling them to learn how to utilize fiction and reading groups as a health‐promoting method. Further research is needed to explore the experiences of teachers, peers, and siblings with using fiction in reading groups as a health promotion intervention, and research is also needed to examine which texts are appropriate.

## Ethics Statement

The study was approved by the Norwegian Agency for Shared Services in Education and Research (Project No. 411733). The public health nurses who contributed as study participants provided informed voluntary written consent to participate in the study, in accordance with the Declaration of Helsinki.

## Consent

The participants of this study did not give written consent for their data to be shared publicly.

## Conflicts of Interest

The authors declare no conflicts of interest.

## Positionality Statement

The research team consisted of public health nurses (first, second, and last authors) and a literary scholar (third author), which is seen as a strength in the preparation of the interview guide, during the interview process and the data analysis.

## Data Availability

Due to the sensitive nature of the research supporting data are not available.
